# Blooming and pruning: learning from mistakes with memristive synapses

**DOI:** 10.1038/s41598-024-57660-4

**Published:** 2024-04-02

**Authors:** Kristina Nikiruy, Eduardo Perez, Andrea Baroni, Keerthi Dorai Swamy Reddy, Stefan Pechmann, Christian Wenger, Martin Ziegler

**Affiliations:** 1 Micro- and Nanoelectronic Systems, Department of Electrical Engineering and Information Technology, TU Ilmenau, Ilmenau, Germany; 2 IHP - Leibniz-Institut fuer innovative Mikroelektronik, Frankfurt/Oder, Germany; 3 BTU Cottbus-Senftenberg, Cottbus, Germany; 4 Chair of Micro- and Nanosystems Technology, Technical University of Munich, Munich, Germany; 5 Institute of Micro- and Nanotechnologies MacroNano, TU Ilmenau, Ilmenau, Germany

**Keywords:** Memristive devices, Neuromorphic computing, Learning from mistakes, Electrical and electronic engineering, Materials for devices, Mathematics and computing

## Abstract

Blooming and pruning is one of the most important developmental mechanisms of the biological brain in the first years of life, enabling it to adapt its network structure to the demands of the environment. The mechanism is thought to be fundamental for the development of cognitive skills. Inspired by this, Chialvo and Bak proposed in 1999 a learning scheme that learns from mistakes by eliminating from the initial surplus of synaptic connections those that lead to an undesirable outcome. Here, this idea is implemented in a neuromorphic circuit scheme using CMOS integrated HfO_2_-based memristive devices. The implemented two-layer neural network learns in a self-organized manner without positive reinforcement and exploits the inherent variability of the memristive devices. This approach provides hardware, local, and energy-efficient learning. A combined experimental and simulation-based parameter study is presented to find the relevant system and device parameters leading to a compact and robust memristive neuromorphic circuit that can handle association tasks.

## Introduction

Perception is a fundamental cognitive ability that enables biological brains to combine partial information into subjectively meaningful overall impressions^1–3^. This is a unique ability of biology, which allows us to perceive our environment and thus to react adequately to it. An ability from which today’s artificial neural networks (ANNs) are far away^4,5^. In particular, this is demonstrated by the fact that biological networks can adapt flexibly and context-dependently to new environmental influences, whereas ANNs can only react to previously trained events^6^. This can essentially be attributed to the rigid network structure used in ANNs, which is trained to perform a defined learning task. Even though ANNs can outperform the human brain in some cases^7,8^, the training process is extremely computationally intensive and results in high resource and energy requirements^9^.

Even if today’s neuroscience is still far away from a unified understanding of the underlying biological mechanisms, it can be shown that biological neuronal networks have different time windows in which there is an increased growth of neuronal synaptic connections and subsequent elimination^10–12^. At this respect, a process called blooming and pruning leads to the extreme efficiency of biological brains^13^. Therefore, in the first years after birth, the human brain creates many more neuronal connections than needed. This number is gradually reduced from around two years of age until adulthood. This has the advantage that a child’s senses tell the brain about its environment and experiences and stimulate neural activity in those areas of the brain relevant for processing^14^. As the amount of input increases over the lifetime, the synapses between the neurons in this area are activated more frequently and those are strengthened, where connections that are little used have a high probability of being eliminated^11,13^. In this context, this process is subject to learning and memory and is believed to be the important precondition leading to our cognitive abilities^15^.

Inspired by the process of blooming and pruning, Chialvo and Bak have proposed in 1999 a model of self-organized learning without positive reinforcement^16^. The learning mechanism of the model selects the most suitable synapse path from a large number of possible ones by pruning synapses that are not used frequently. Thus, learning occurs through mistakes (negative feedback), with the network topology adaptively adjusting as the environment and brain demand change^16–19^. In a recent simulation work, it was mentioned that neuromorphic networks using memristive devices enable the Chialvo–Bak model to be implemented in hardware with very little peripheral computation overhead^20^. In particular, this is due to the fact that the Chialvo–Bak model serves the intrinsic properties of memristive devices and enables, therefore, the realization of robust and fault-tolerant neuromorphic systems^16,20^. This includes the intrinsic stochastic of the memristive devices and their integration into a suitable network computing scheme^3,21,22^.

Here, we implement the learning from mistakes inspired by Chialvo and Bak model in a two-layer neural network based on memristive connections realized in a CMOS-integrated HfO_2_-based resistive random-access memory (RRAM) structured in a 4 kbit array. For training the actual output value of the network is compared with the desired output and those cells of the memristive array are suppressed which do not contribute to the desired output. The pruning mechanism uses reset voltage pulses to put the selected memristive cell into a less conductive state with a certain distribution. This inherent stochasticity of the memristive cells in the RRAM array is exploited to achieve convergence of the neural network. The experimental results are supported by simulations that determine the range of parameters for the neural network. To demonstrate the potential of the network, we apply the system to learning facial expressions to interpret emotions. We show how facial expressions can be linked using this network to represent emotions such as anger, fear, disgust, joy, sadness, and surprise.

**Figure 1:**
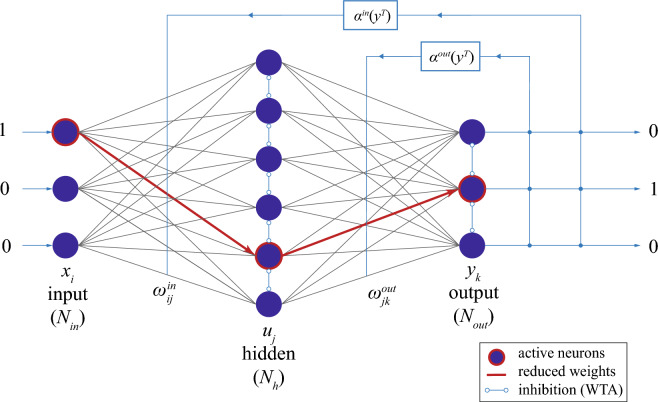
Schematic of the implemented memristive neural network which consists of two fully connected layers. Each connection shown is represented by a memristive device whose resistance state determines the respective coupling strength. Neurons in the hidden and output layer are lateral inhibitory coupled. Therefore, a winner-take-it-all (WTA) algorithm is used so that in each iteration step only the neuron that receives the highest input current is active. This means that only one neuron is active per layer and iteration step. Thus the active path is used for the learning mechanism. If the active path does not lead to the desired pattern at the output, the memristive devices of the path are reset via pruning voltage pulses, which define the respective learning rates $\alpha ^{in}$ and $\alpha ^{out}$ of the input and output layer, respectively.

## Results

### The memristive network learning scheme

The network scheme used is shown in Fig. 1 and consists of two fully connected neuron layers. The core idea of the learning scheme goes back to Chialvo and Bak^16^ proposing a learning scheme that learns from mistakes. This means that those synaptic connections that do not show the desired output value are reduced. Thus, in each iteration step, the learning algorithm determines the pathway that makes the maximum contribution to the output, and if the current output does not reflect the desired output, the associated synaptic connections are depressed.

Motivated by this idea, in the present work we constructed a local learning rule for the weight update process by employing the Hebbian learning theory^23,24^, which can be adapted to memristive devices^25–27^. Here, the learning rule for the weight update of the input $\omega _{ij}^{in}$ and output layer $\omega _{jk}^{out}$ can be written as:

1 $$\begin{aligned} \frac{d\omega _{ij}^{in}}{dt} = \alpha ^{in}\left( V,y^T_k\right) \cdot x_i \cdot u_j \end{aligned}$$

2 $$\begin{aligned} \frac{d\omega _{jk}^{out}}{dt} = \alpha ^{out}\left( V,y^T_k\right) \cdot u_j \cdot y_k \end{aligned}$$

where $\alpha ^{in,out}$ are voltage-dependent adaptive learning rates whose values depend on the desired output value $y^T_k$.

3 $$\begin{aligned} \begin{matrix} \alpha ^{in,out}(V,y^T_k) &{}= &{} &{} \biggl \{ \begin{matrix} g^{in,out}(V) &{} \text{ if } \quad y_k \ne y^T_k \\ 0 &{} \text{ otherwise } \end{matrix} \end{matrix} \end{aligned}$$

here $g^{in,out}(V)$ defines the reset voltage pulses used for depressing the respective synaptic connections. It thus controls the changes in the weights of the respective synaptic and determines the learning success of the network with respect to its learning task. Furthermore, $x_i$, $u_j$, and $y_k$ are, respectively, the neurons of the input, middle, and output layers which are realized by binary neurons (Fig. 1). While the activity of input neurons $x_i$ corresponds to the binary input values, which are either 1 or 0, the activities of the hidden and output neurons $u_j$ and $y_k$ are implemented via

4 $$\begin{aligned} \begin{matrix} u_j &{} = &{} &{} \Biggl \{ \begin{matrix} 1 &{} \text{ if } \max \limits _{ j} \biggl ( \frac{1}{N_{in}}\sum \limits _{i=1}^{N_{in}} \omega _{ij}^{in} \cdot x_i \biggr ) \\ 0 &{} \text{ otherwise } \end{matrix} \end{matrix} \end{aligned}$$

and

5 $$\begin{aligned} \begin{matrix} y_k &{} = &{} &{} \Biggl \{ \begin{matrix} 1 &{} \text{ if } \max \limits _{ k} \biggl ( \frac{1}{N_{H}}\sum \limits _{j=1}^{N_H} \omega _{jk}^{out} \cdot u_j \biggr ) \\ 0 &{} \text{ otherwise } \end{matrix} , \end{matrix} \end{aligned}$$

where $N_{in}$ and $N_H$ are the numbers of neurons in the input and hidden layer, respectively. Furthermore, a lateral inhibition within the neural layer is introduced within the neuron model by a winner-take-all (WTA) learning scheme, in which only the most active neuron of a layer is considered and all other neurons of the layer are set to inactive (cf. red framed neurons Fig. 1). Thus, the key parameter for the implementation of the learning scheme is the reset dynamics of the memristive devices as well as their variability and initial state. Therefore, in the following, we will take a closer look at the memristive elements used in the implementation and how they fit into the learning scheme.

**Figure 2:**
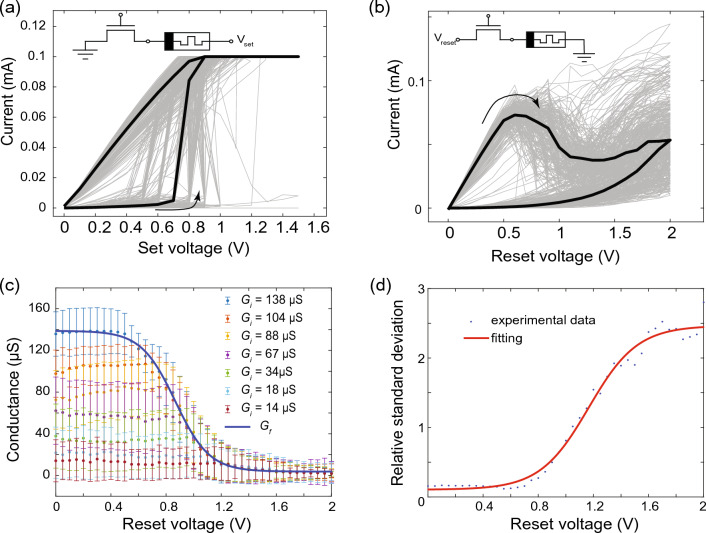
Memristive device characteristics. Current-voltage (I–V) curves of used memristive devices (gray) and median curve (black). (**a**) The setting of the device to a low resistant state and (**b**) resetting to its high resistant state. Insets: measurements schematic of the used CMOS-integrated HfO_2_-based memristive devices which are organized in a 1T-1R cell within a 64 $\times$ 64 memory array. (**c**) Final conductance dependence on pruning voltage (dots) and fitting curve (blue line) which describes the reset process. For the reset voltage pulse different voltage amplitudes and a constant pulse width of $\Delta t =100ms$ have been used. Experimental conductance is obtained by applying the voltage pulse to the memristor with the initial conductance $G_i$. (**d**) The relative standard deviation of final state $D=\sigma G/G$ on voltage: experiment (blue dots) and fitting (red line)).

As memristive devices, CMOS-integrated HfO_2_-based RRAM devices fabricated in a 130 nm CMOS technology were employed, which are integrated into 4 kbit memory arrays organized in 64 $\times$ 64 1T-1R cells (see methods part for further details). Before the cells can be switched, an electroforming step is required. For this purpose, the incremental step pulse with verification algorithm (ISPVA) was used^28^. After electroforming, the memristive cells of the memory array switched as shown in Fig. 2. In Fig. 2a,b, representative *I*-*V* curves of the memristive devices are shown, while in the insets to Fig. 2a,b a schematic of the device structure and its configuration as a 1T-1R cell is given. The selector device is an NMOS transistor connected in series with a memristive cell consisting of a TiN/Ti/HfO_2_/TiN layer sequence. Depending on the desired voltage polarity for the set, reset, and read operations, either a positive voltage is applied to the source terminal of the transistor or to the top contact of the memristive device stack, while the other terminal is grounded. This means that the memristive cells are set when a positive voltage (see V_set_ in Fig. 2a) is applied to the top contact of the memristive stack, while the memristive cell is reset when a positive voltage (see V_reset_ in Fig. 2b) is applied to the source terminal of the transistor. For the *I*–*V* curves, the voltage was varied from 0 to 1.5 V to set the memristive cells, while a voltage sweep from 0 to 2 V was used to reset the devices. As you can see, the devices show a bipolar switching with an abrupt change in resistance from the initial high resistance state (HRS) to the low resistant state (LRS) at about 0.7 V. Functionally, the switching mechanism is due to the formation of filaments from oxygen vacancies, as described for these memristive devices in^22^.

To use the cells for the learning scheme proposed here, the cells were set from their high resistive state (HRS) to their low resistive state (LRS). The transition, which is then important for the successful training of the network, is carried out by resetting the memristive cells. However, the value of the final conductance in the HRS depends on both the applied voltage *V* and the initial (obtained) conductance value $G_i$ in the LRS, as shown in Fig. 2c for seven different conductance values in the LRS ranging from 14 to 138 $\upmu$S. To describe this process in more detail and to be able to investigate it systematically for the application, we describe the memristive cells using the general memristor equations^29,30^:

6 $$\begin{aligned} I = G(V,t) \cdot V \end{aligned}$$

and

7 $$\begin{aligned} \frac{dG(V,t)}{dt} = f(V,t) \end{aligned}$$

Here, *f*(*t*, *V*) is a time and voltage-dependent function that depends on the underlying switching process of the memristive device^27,29^. For a constant voltage pulse width $\Delta t$ of the pruning pulse, this function defines the pruning conditions via eq. 3, i.e. $g^{in,out}(V)\propto f(V,\Delta t$).

In order to systematically describe the voltage-dependent reset dynamics without going into detail about the physical switching process, we used the following relationship for the final conductance $G_f$ after the application of a pruning pulse of constant width and varying amplitude:

8 $$\begin{aligned} G_f(V) = G_{ON}\cdot k(V)+G_{OFF}\cdot (1-k(V)) \end{aligned}$$

where $G_{ON}$ and $G_{OFF}$ are conductance in LRS and HRS accordingly, *k*(*V*) describes the voltage dependency of the conductance change. From experiment, the conductance after low voltage pulse should be $G_{ON}$, which leads to *k*(0*V*) close to 1, for high voltage pulse should be $G_{OFF}$, which leads to *k*(1.5*V*) close to 0. Such behavior we describe, via a logistic equation according to:

9 $$\begin{aligned} \frac{dk}{dV} = \frac{1}{\Delta V} \cdot k\cdot (1-k) \end{aligned}$$

This equation describes a sigmoid curve, that is also observed in experiment (Fig. 2c) and can be solved by

10 $$\begin{aligned} k(V) = \frac{1}{1 + exp\left[ -\frac{(V-V_0)}{\Delta V}\right] } \end{aligned}$$

where $V_0$ and $\Delta V$ are fitting parameters describing the dynamics of the conductance change during the reset process of the memristive device.

The voltage dependency of $G_f(V)$ according to Eqs. 7–10 (solid line) is compared in Fig. 2c to experimental data (dots). Here, reset pulses, named as pruning pulses in the following, of voltage amplitudes ranging from 0 V to 2 V and a constant pulse width of 100 ms were applied to previously set memristive devices in different initial conductance states $G_i$. The experimental data were measured at a read voltage of 0.1 V. For an initial conductance $G_i=138 \mu S$ a with the model predicted $G_f(V)$ curve is added as blue line to Fig. 2c. The fitting constants were determined here as $V_0 = 0.85V$ and $\Delta V = 0.16V$. As can be seen from Fig. 2c, the chosen approach shows good agreement with the experimental conductance change.

Important for the learning scheme used here is that for voltage pulses smaller than 1 V the memristive cells are not completely reset and a clear variability in the conductance values is present in the HRS, which we will examine in more detail later in connection with the learning scheme. This relative variability is calculated as $D=\sigma G/G$ where $\sigma G$ is the standard deviation of the conductance states *G* and can be also described in connection to pruning pulse by:

11 $$\begin{aligned} D(V) = D_{OFF} + \frac{D_{ON} - D_{OFF}}{1+ exp\left[ -\frac{(V-V_D)}{\Delta V_D}\right] } \end{aligned}$$

with the fitting parameters $V_D = 1.16V$, $\Delta V_D = 0.18V$ a good agreement with experimental data is achieved (cf. Fig. 2d). The described model was used for the simulation of the learning scheme which is functionally based on memristive devices.

### Network performance

Based on the memristive network learning scheme described, a network with, respectively, six input neurons ($N_{in} = 6$) and six output neurons ($N_{out} = 6$), as well as 18 neurons in the hidden layer ($N_{h} = 18$) was realized. Thus, a total number of 216 memristive devices were employed, which are divided between the two layers of the network. For the hardware implementation, the in ref.^22^ presented ANN board was used which connects a 4-kbit memristive crossbar array to a computer interface containing the learning algorithm (see methods).

**Figure 3:**
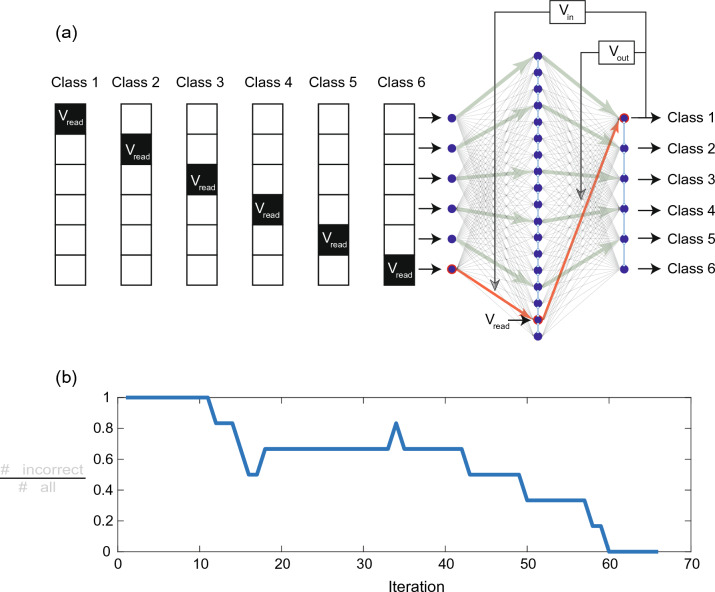
Learning from mistakes in the experiment. (**a**) Learning methods for classification of vectors (test task). (**b**) Training phase: error as the normalized number of incorrect outputs on learning iteration.

For the demonstration of the functionality of the learning scheme and to investigate the behavior of the memristive devices within the network, we first gave the network the testing task shown in Fig. 3. For this, we have created six sparse input patterns, which the network should assign to six different classes. Each of the patterns contains a single entry “1” and five “0” input values, as sketched in Fig. 3a. These patterns we use for training and testing. For the experimental investigation, a positive voltage pulse (voltage at the top contact of the device stack as in Fig. 2a) is used for the entry “1”, while for the “0” values of the pattern, no voltage pulse was applied to the network. For the applied voltage pulse a voltage amplitude was chosen below the threshold voltage not to change the device resistance, referred to as read pulse $V_{read}$ in the following. During learning at each iteration step one of the shown pattern, i.e. a single voltage pulse $V_{read}$, was applied to the network via the neurons of the input layer, while the resulting activity of the output layer neurons $y_k$ were recorded and compared to the desired target value $y_k^T$ expected for the given input pattern. If the obtained output pattern $y_k$ was not consistent with the desired one $y_k^T$, the pruning algorithm has been used. The WTA rule, i.e. the lateral inhibition of the neurons in the hidden layer according to Eqs. 4 and 5, sets that neuron of the hidden layer with the highest input to “1”, while all others were set to “0”. This means, for the active neuron a read voltage $V_{read}$ was applied, and if the resulting output pattern $y_k$ was incorrect, pruning voltages (reset pulses), labeled as $V_{in}$ and $V_{out}$, were applied to the neurons of the input and output layers, respectively. The corresponding memristive devices of the path were rested eliminating the wrong path for connection between input and output layers (red arrows in Fig. 3a). In the case of successful learning (here we call learning run successful if it reaches 100 percent of accuracy), only those paths remain, which are leading to the desired connectivity between input and output neurons of the network, as sketched by green arrows in Fig. 3a. In order to evaluate the learning progress, we determined the error of the network per iteration step, which has been calculated by dividing the incorrect output values for all of the patterns in each iteration step by the number of input patterns. The therewith-obtained evolution of the error is shown in Fig. 3b. As can be seen, the network was able to learn the task within 60 iterations.

**Figure 4:**
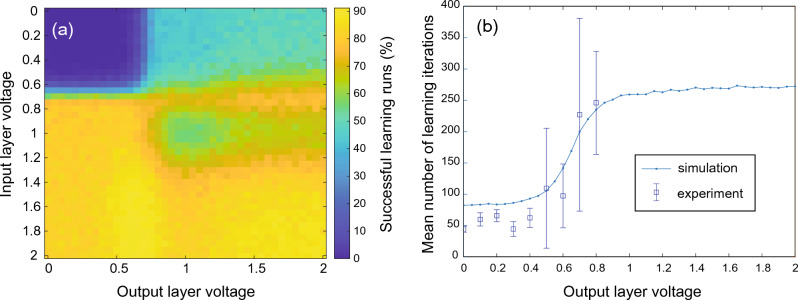
Parameter screening for finding the optimal performance of the learning task of Fig. 3. Therefore, the influence of the pruning voltages of the input and output layers on system performance was investigated: (**a**) successful learning runs in dependence on input-output pruning voltage combinations. (**b**) Dependence of the mean number of learning iterations on the pruning voltage of the output layer for a fixed input layer pruning voltage of 1 V. Simulation data are compared in (**b**) with experimental data.

To find out the optimal network size for the given problem, a fine-tuning of the network parameters is necessary. In the following, a procedure developed for this purpose is presented, which uses the simulation model of the memristive devices described above. The data obtained from the parameter screening are summarized in Fig. 4. We found, that the number of learning iterations for achieving successfully the learning task can vary significantly (see Fig. 4). For the parameter study, the maximum number of learning iterations was set to 10000, and 1000 different learning runs were performed using the same initial conditions for each pruning voltage pair $V_{in}$ and $V_{out}$. The results are given as a heating map in Fig. 4a which shows the number of successful learning runs per pruning voltage pair. A maximum of successful learning runs has been achieved for pruning input voltages $V_{in}$ ranging from 1.5 to 2.0 V, while the pruning voltage required for the output layer $V_{out}$ in that voltage interval does not influence the performance of the network significantly. Hence, the pruning mechanism for the memristive devices of the input layer seems to have a higher sensitivity to successfully learning the task, while specific connectivity of the output layer might be less important for learning.

To analyze this finding in more detail, we have calculated the mean number of iteration steps necessary for learning the task. Therefore, only those of the 1000 learning runs have been counted which were successful. In Fig. 4b the obtained simulation results are compared with experimental data. First, we found that if a learning run is successful, the network has learned the task in the first 250 learning iterations. Second, we found that the required iteration number for learning is strongly decreased by using pruning voltages for the memristive devices in the output layer between 0 and 0.4 V. Furthermore, we found from our experimental investigation that for such small output pruning voltages the variability between different learning runs drastically decreases (see arrow bars in Fig. 4b obtained from five learning runs for each voltage value). This result suggests that the initial random connection of neurons from the hidden to the output layer might be sufficient to cope with the learning task. This finding, however, gives particular benefit to the distribution of the resistant states of the memristive devices which we like to analyze in further detail.

**Figure 5:**
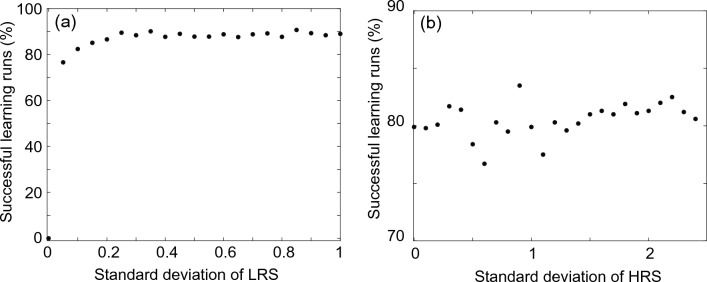
Influence of device variation on system performance. Percentage of achieved learning runs on the standard deviation of (**a**) LRS and (**b**) HRS.

To study the network performance as a function of the variability of the resistive states of the memristive devices in more detail, we have performed simulations for different standard deviations in a wide range. The therewith-obtained rates of successful learning runs are plotted as a function of the resistant distributions of the memristive devices for the LRS and HRS in Fig. 5a, b, respectively. For this set of simulations, the pruning voltage for the memristive devices in the output layer was set to zero, while an amplitude of 2 V was used as the pruning voltage for the input layer memristive devices. According to our experimental investigation of the variability in the rest characteristic of the memristive devices for different pruning voltages, shown in Fig. 2d and described by Eq. 9, a relative variability of $\sigma = 0.1$ for pruning voltages below 0.4 V and a $\sigma = 2.5$ for pruning voltages larger then 1.6 V can be assumed. This is due to the fact that at very small pruning voltage amplitudes the devices are not reset to the HRS completely and thus the variability of the devices is determined by the variability of the LRS. Thus, for the results shown in Fig. 5a, we have assumed a variability of the LRS up to $\sigma = 1$. What we can see in Fig. 5a is that in this range the learning performance of the system is not affected by the device variability. On the contrary, if the variability is too low or if there is no variability between the memristive devices, the learning performance is poor or zero (see data point for $\sigma = 0$ in Fig. 5a). This finding can be explained by the fact that due to the use of lateral inhibition between neurons in the hidden layer, introduced by the WTA mechanism, a certain variability of the devices is needed for operation. To complete the picture, we also took a closer look at the distribution of the HRS of the memristive devices (see Fig. 5b), where a higher dispersion was measured experimentally. But, also here we found that the learning mechanism benefits from the variability between the resistant states of the memristive devices in the high ohmic range (HRS). This particular proves that we can use and benefit from a relatively wide distribution of the resistant states of the memristive devices.

### Associative learning: interpretation of emotions by learning facial expressions

An essential feature for learning processes in biology is the exchange with the environment that leads to the continuous adaptation and modification of the connectivity in neuronal networks^16^. This particularly leads to the association of different information to a coherent perception and in a way that previously unrelated information is connected to a uniform representation. In order to show in which way such a process (referred to associative learning) can be emulated with the network realized here, we have considered the learning of facial expressions as an example of the perception of emotions.

**Figure 6:**
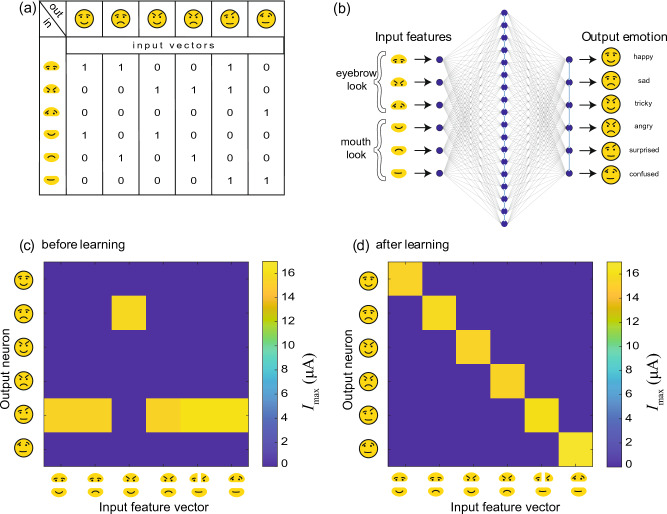
Learning from mistakes in the experiment. (**a**) Input features and output emotions and corresponding input vectors. (**b**) The network scheme including such input as eyebrow and mouth look which lets the system understand which emotion the face expresses. The experimental current output normalized to max output for different neurons and input patterns: (**c**) before learning and (**d**) after learning.

A schematic representation of the network structure implemented in hardware to emulate the learning of facial expressions is shown in Fig. 6a,b. The network consists respectively of six input and six output neurons, and 18 neurons in the hidden layer. This results in 216 memristive devices in the input layer and output layer, respectively. As a learning task, we have presented six different combinations of the mouth parts (mouth look) and eye parts (eyebrow look) as feature vectors, shown in Fig. 6a. For emotion recognition, these vectors must be associated by the network with a particular emotion. Each of these feature vectors has therefore six entries that must be linked to six different emotions expressing the emotions happy, sad, tricky, angry, surprised, and confused. Thus, this task requires linking multiple features of an object to its class and is a suitable problem for the presented version of the Chialvo–Bak model, as sketched in Fig. 6b.

Before learning the neurons are randomly connected with a Gaussian weight distribution of $0.1G_{on}$. Therefore, the memristive devices of the CMOS-integrated crossbar array have been set from the HRS to a LRS. Here, a set voltage of 2V has been applied to the individual cells of the memristive crossbar array which leads to the desired resistant distribution. By applying thereafter the defined feature vectors to the network, an incorrect interpretation of the facial information is obtained prior to learning, as shown by the connectivity matrix in Fig. 6c. In the presented connectivity matrix the respective output neuron with the highest current value, calculated via Eq. 5, is plotted as a function of the input feature vectors and output neurons. Here you can see that prior to learning five of the six different feature vectors can only be assigned to one facial expression (surprised). After learning the connectivity matrix has changed, as shown in Fig. 6d. Now each feature vector is assigned exactly to one of the emotions.

For the learning process, we used the previously found values for the pruning voltages, i.e. $V_{in} = 1.0V$ and $V_{out} = 0V$ for the input and output layer, respectively. We found that the learning procedure was more protracted than in the simpler cases considered above. On average, 130–140 iterations were needed for the system to learn, with the number of successful learning runs dropping to 60$\%$.

**Figure 7:**
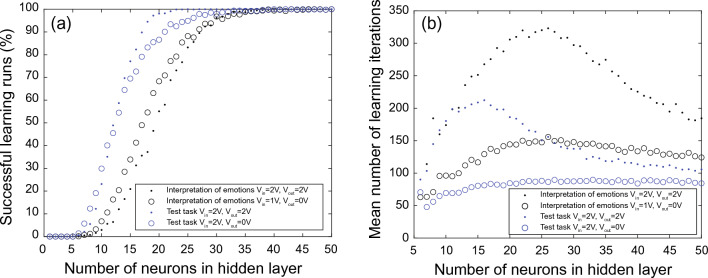
Parameter screening for finding the optimal network size. (**a**) Percentage of successful learning runs and (**b**) mean number of learning iterations on size of the hidden layer.

For the Chialvo–Bak model, it is known that the number of successful learning runs depends on the number of neurons in the hidden layer^18–20^. Therefore, the number of neurons in the hidden layer should be taken into relation to the complexity of the task. To do so, another set of simulations has been performed in which the number of hidden neurons has been increased up to $N_{h} = 50$. Figure 7a shows the results for successful learning runs, Fig. 7b for mean number of learning iterations. We found that for a higher success rate, it is necessary to increase the number of neurons in the hidden layer from $N_{h} = 18$ to $N_{h} = 35$ (Fig. 7a). Furthermore, the comparison with the previously discussed test task shows that the number of hidden layer neurons depends on the complexity of the task (see Fig. 7)). In the simpler task, $N_{h} = 18$ was already sufficient to cope with task complexity, while a more complex learning task requires more neurons in the hidden layer. In general, we found evidence that with a sufficiently high number of neurons in the hidden layer, it is possible to achieve high learning success rates even with non-optimal network parameters, as shown in Fig. 7a. For that learning runs pruning voltages of $V_{in} = 2.0V$ and $V_{out} = 2.0V$ have been chosen for the input and output layer, respectively, which shows not optimal learning conditions in the made parameter tuning study shown in Fig. 4. Our finding is consistent with previous findings^20^ which shows that with a large enough number of neurons in the hidden layer any input pattern can be successfully learned by the network. This finding is particularly interesting since it avoids complicated parameter tuning of the network and provides a relatively robust network learning scheme. On the other hand, this can also lead to large networks that are technically difficult to implement. Therefore, a compromise must be found between network size and flexibility in order to estimate the necessary parameter-tuning effort.

## Discussion

A robust memristive learning scheme was demonstrated that does not require positive reinforcement, uses self-organization, exploits the inherent stochasticity of memristive devices, and is inspired by the learning from mistake algorithm proposed by Chialvo and Bak. The latter uses one of the most important adaptation mechanisms of biological brains during their development in the first years of life, known as blooming and pruning. Here, those synaptic connections are eliminated from a multitude of connections that are not used frequently, i.e. those connections are eliminated that do not correspond to the desired result. This allows neural networks to adapt their topology to different changing environments in a flexible and situation-dependent way. In contrast to standard training techniques, such as backpropagation, the proposed algorithm is easily implementable in hardware and doesn’t require high accuracy of switching.

The described properties we have technically transferred here to a two-layer memristive neural network. For this purpose, we have used the reset dynamics of CMOS integrated HfO_2_-based memristive crossbar arrays, which we have investigated experimentally and with the help of a simulation model. We were able to show that the variability of the resistance states due to the resetting of the memristive devices leads to convergence of the learning algorithms and finding optimal voltage amplitudes for the pruning pulses through a thorough parameter study. Furthermore, we could show that we can realize a relatively compact and robust two-layer neural network that can already handle simple association tasks, i.e. the recognition of simple facial expressions as an example for the interpretation of emotions. For more complex tasks, it is possible to use the pattern directly as input to the network or to pre-process the data to extract the features and then use these features as input to the learning. In this way, learning from mistakes provides a tool for classification in a fully hardware network with a simple training phase.

In addition, we discussed to what extent the number of neurons in the hidden layer makes a detailed parameter-tuning nursery and were able to show that the exact setting of the voltage parameters for pruning is less important for sufficiently large networks. This is in agreement with the work by Chialvo et al. and shows a possible way for self-organized neural networks, which exploit the unique properties of memristive devices in order to achieve a new degree of freedom in the technical emulation of complex biological learning processes.

## Materials and methods

### Device fabrication

The memristive cells of the crossbar structure consist of sputtered 150 nm thick top and bottom TiN electrodes, a sputtered Ti layer with a thickness of 7 nm, and an 8 nm thick HfO_2_ layer deposited by atomic layer deposition (ALD). The devices were integrated into 4-kbit memory arrays organized in a 64 $\times$ 64 1T-1R cell configuration. The 1T-1R memory cell consists of an NMOS transistor, serving as a selector, fabricated in 130 nm CMOS technology with its drain connected in series with the memristive cell. The area of the memristive device was defined as 0.4 $\upmu$m^2^. More details about devices are in ref.^31,32^.

### Hardware implementation

The learning scheme was implemented with CMOS-integrated HfO_2_-based memristive devices packaged into a 4 kbit array. Therefore in ref.^22^ described ANN board was used in which the packaged 4 kbit array was connected to a printed circuit board (PCB) using a standard 64-pin integrated circuit (IC) socket. The PCB contains a microcontroller (Arduino Mega 2560) which addresses the pins of the memristive array and provides an interface to a conventional computer on which the algorithm runs. To simulate the neurons and control the complete experimental setup a MatLab code was developed. The read-out and pruning pulses were applied using a Keysight B2902A source measurement unit.

## Data Availability

The datasets generated and analysed during the current study are available from the corresponding author on reasonable request.
